# The N-terminus acts as a lever to support amphetamine-induced substrate efflux by the serotonin transporter

**DOI:** 10.1186/2050-6511-13-S1-A54

**Published:** 2012-09-17

**Authors:** Sonja Sucic, Carina Kern, Harald H Sitte, Michael Freissmuth

**Affiliations:** 1 Institute of Pharmacology, Center for Physiology and Pharmacology, Medical University Vienna, 1090 Vienna, Austria

## Background

We have previously shown that a highly conserved threonine at position 81, in the amino terminus of SERT, plays a key role in SERT function, by driving the transporter into a state that supports amphetamine-induced efflux [1]. Truncation of the first 64 amino acids or tethering the N-terminus to an additional transmembrane helix both abolish amphetamine-induced efflux by SERT [1].

## Methods

Alanine scanning mutagenesis was carried out along the N-terminal region of SERT to pinpoint the residues involved in maintaining amphetamine-induced efflux. Two residues at a time were replaced by alanine using a site-directed mutagenesis kit (Quikchange kit, Stratagene). The mutants were characterised by uptake, release and binding assays; surface expression was visualised by confocal microscopy. Conformational sensitivity of the N-terminus was examined by proteolytic cleavage. Tryptic digestion of membranes prepared from SERT-expressing cells was performed under different buffer conditions, in the absence or presence of various ligands, and detected by Western blotting.

## Results

Although all mutant SERTs generated in this study were targeted to the plasma membrane, some exhibited dramatic reductions in amphetamine-induced efflux. Moreover, they were all active with respect to [^3^H]serotonin uptake, showing no marked changes in the affinity or velocity of substrate uptake. The reduction in efflux did not result from impaired affinity of the mutants for amphetamines (shown by [^3^H]imipramine binding assays). In outward-facing conformations of SERT, the N-terminus is less susceptible to proteolytic digestion, possibly because it is shielded upon accompanying structural rearrangements. In inward-facing states, it is less susceptible to cleavage only when amphetamines are bound.

## Conclusions

The region encompassing residues 22–32 may be a pivot for the movement of the N-terminus allowing amphetamine-induced release to occur. The mutagenesis and proteolysis data are consistent with the N-terminus of SERT acting as a lever, promoting substrate release by a second moiety of the oligomer.
